# Supplementary material for: An Evaluation of the United Kingdom Motor Neuron Disease Nurses and Allied Health Professionals (UK MND NAHP) Workforce: A Census
Source: PLoS One. 2025 Jul 11;20(7):e0319628. doi: 10.1371/journal.pone.0319628 (PMC12250277; doi:10.1371/journal.pone.0319628)
Supplement: S1 Table — n, sample size; %, percentage. (DOCX) [file pone.0319628.s001.docx]

**S1 Table*:* Summary table of participant demographics.**

| **Sex Distribution** | **n (%)** |
| --- | --- |
| Male | 5 (7.80) |
| Female | 59 (92.20) |
| **Age Ranges** |  |
| 18-25 | 1 (1.56) |
| 26-30 | 5 (7.81) |
| 31-35 | 6 (9.38) |
| 36-40 | 8 (12.50) |
| 41-45 | 10 (15.63) |
| 46-50 | 13 (20.31) |
| 51-55 | 10 (15.63) |
| 56-60 | 10 (15.63) |
| 61-65 | 1 (1.56) |
| **Ethnicity** |  |
| Indian | 1 (1.56) |
| Other White Background | 1 (1.56) |
| White British | 62 (96.88) |
| **Highest qualification achieved** |  |
| Bachelor of Nursing/Bachelor of Sciences (BN/BSc) | 22 (34.38) |
| Higher Education Diploma (HE Dip) | 3 (4.688) |
| Masters of Nursing/Masters of Sciences (MN/MSc) | 17 (26.56) |
| Other/ Other Allied Health Professional Qualification | 8 (12.50) |
| Registered Nurse Diploma (RN Dip) | 14 (21.88) |
| **Job title** |  |
| Allied Health Professional (AHP) | 4 (6.250) |
| Clinical Nurse Specialist (MND) | 32 (50.00) |
| Clinical Nurse Specialist (non-MND specific) | 3 (4.690) |
| Clinical Research Nurse | 10 (15.63) |
| MND Care Service Coordinator | 15 (23.44) |

n, sample size; %, percentage
