# Supplementary material for: An Evaluation of the United Kingdom Motor Neuron Disease Nurses and Allied Health Professionals (UK MND NAHP) Workforce: A Census
Source: PLoS One. 2025 Jul 11;20(7):e0319628. doi: 10.1371/journal.pone.0319628 (PMC12250277; doi:10.1371/journal.pone.0319628)
Supplement: S2 Table — A. Qualification by Clinical Nurses (MND and non-MND specific). MND, Motor Neuron Disease; n = sample size. B. Qualifications by AHP. AHP, Allied Health Professional; MN/MSc, Masters of Nursing/Masters of Sciences; BN/BSc, Bachelor of Nursing/Bachelor of Sciences; n, sample size; %, percentage. (DOCX) [file pone.0319628.s002.docx]

**S2 Table. Qualification Demographic.**

**S2A Table. Qualification by Clinical Nurses (MND and non-MND specific).**

| **Clinical Nurses (MND and non-MND specific)** | **n** |
| --- | --- |
| Bachelor of Nursing/Bachelor of Sciences (BN/BSc) | 13 |
| Higher Education Diploma (HE Dip) | 3 |
| Masters of Nursing/Masters of Sciences (MN/MSc) | 8 |
| Other/ Other Allied Health Professional Qualification | 2 |
| Registered Nurse Diploma (RN Dip) | 9 |

MND, Motor Neuron Disease; n, sample size

**S2B Table. Qualifications by AHP.**

| **AHP** | **n** |
| --- | --- |
| Masters of Nursing/Masters of Sciences (MN/MSc) | 1 |
| Other/ Other Allied Health Professional Qualification | 2 |
| Bachelor of Nursing/Bachelor of Sciences (BN/BSc) | 1 |

AHP, Allied Health Professional; MN/MSc, Masters of Nursing/Masters of Sciences; BN/BSc, Bachelor of Nursing/Bachelor of Sciences; n, sample size; %, percentage
