# Supplementary material for: An Evaluation of the United Kingdom Motor Neuron Disease Nurses and Allied Health Professionals (UK MND NAHP) Workforce: A Census
Source: PLoS One. 2025 Jul 11;20(7):e0319628. doi: 10.1371/journal.pone.0319628 (PMC12250277; doi:10.1371/journal.pone.0319628)
Supplement: S3 Table — Employment Demographics. A. Country of Work by Clinical and Non-clinical HCP. HCP, Healthcare Professional; n = sample size; % = percentage. B. Clinical vs Non-clinical and Full-time vs Part-time HCP. HCP, Healthcare Professional; n = sample size; % = percentage. C. Time of work per week (hours), Worked in MND care (months), Number of Patients (n), Time in Current Post (weeks). MND, Motor Neuron Disease; n = sample size; % = percentage. D. Vacancies in Core MDT. MDT, Multidisciplinary Team; n = sample size; % = percentage. (DOCX) [file pone.0319628.s003.docx]

# **S3 Table. Employment Demographics.**

**S3A Table. Country of Work by Clinical and Non-clinical HCP.**

|  | **Total** | **Clinical** | **Non-clinical** |
| --- | --- | --- | --- |
| **Country of work** | **n (%)** | **n (%)** | **n (%)** |
| England | 35 (54.69) | 20 (51.28) | 15 (60.00) |
| NA | 3 (4.69) | 2 (5.13) | 1 (4.00) |
| Other | 3 (4.69) | 2 (5.13) | 1 (4.00) |
| Scotland | 23 (35.94) | 15 (38.46) | 8 (32.00) |
| **Grand Total** | **64 (100.0)** | **39 (100.0)** | **25 (100.0)** |

HCP, Healthcare Professional; n, sample size; %, percentage

**S3B Table. Clinical vs Non-clinical and Full-time vs Part-time HCP.**

| **Clinical vs non-clinical** | **Full Time (n (%))** | **Part Time (n (%))** | **Sub-Total (n (%))** |
| --- | --- | --- | --- |
| Clinical | 22 (56.41) | 17 (43.59) | **39 (60.94)** |
| Non-Clinical | 13 (52.00%) | 12 (48.00) | **25 (39.06)** |
| **Grand total** | **35 (54.69%)** | **29 (45.31)** | 1. **(100.0)** |

HCP, Healthcare Professional; n, sample size; %, percentage

**S3C Table. Time of work per week (hours), Worked in MND care (months), Number of Patients (n), Time in Current Post (weeks)**.

|  | **Hours Worked (hours)** | **Worked in MND care (Months)** | **Number of Patients (n)** | **Time in Current Post (weeks)** |
| --- | --- | --- | --- | --- |
| Mean | 31.81 | 112.5 | 94.87 | 343.4 |
| Min | 15 | 3 | 1 | 3 |
| Max | 37.50 | 360 | 380 | 1144 |
| Standard Deviation | 7.256 | 89.01 | 94.30 | 286.8 |
| Median | 37.50 | 84 | 45 | 260 |

MND, Motor Neuron Disease; n, sample size; %, percentage

**S3D Table. Vacancies in Core MDT**.

| **Vacancies in Core MDT (Clinical workers only)** | **n** | **%** |
| --- | --- | --- |
| Yes | 11 | 28.21 |
| No | 26 | 66.67 |
| Not Applicable | 2 | 5.13 |
| **Grand total** | **39** | **100.0** |

MDT, Multidisciplinary Team; n, sample size; %, percentage
