# Supplementary material for: An Evaluation of the United Kingdom Motor Neuron Disease Nurses and Allied Health Professionals (UK MND NAHP) Workforce: A Census
Source: PLoS One. 2025 Jul 11;20(7):e0319628. doi: 10.1371/journal.pone.0319628 (PMC12250277; doi:10.1371/journal.pone.0319628)
Supplement: S4 Table — Clinical Scoring. A. Competencies of MND care based on the NICE guidelines (2016) and NHS MND Advanced Clinical Nurse Specialist Pillars of Practice (2019). MND, Motor Neuron Disease; NICE, National Institute for Health Care and Excellence; NHS, National Health Service; MDT, Multidisciplinary Team; n, sample size; %, percentage. B. HCP/ Specialty Involvement in Core MDT. HCP, Healthcare Professional; MDT, Multidisciplinary Team; n = sample size; % = percentage. C. Established Relationship with NHS Services. NHS, National Health Service; AAC, Augmented and Alternative Communication; n, sample size; %, percentage. (DOCX) [file pone.0319628.s004.docx]

**S4 Table*:* Clinical Scoring**

**S4A Table. Competencies of MND care based on the NICE guidelines (2016) and NHS MND Advanced Clinical Nurse Specialist Pillars of Practice (2019).**

| **Competencies of MND Care** | **Yes (n (%))** | **No (n (%))** |
| --- | --- | --- |
| Provision of Information and Expertise | 38 (97.44) | 1 (2.56) |
| Advocate and Link for the Patient and their Family with the wider MDT | 36 (92.31) | 3 (7.69) |
| Cognitive Screening | 29 (74.36) | 10 (25.64) |
| Respiratory Assessment | 24 (61.54) | 15 (38.46) |
| Nutrition Assessment | 31 (79.49) | 8 (20.51) |
| Anticipatory Care Planning | 35 (89.74) | 4 (10.26) |
| Muscle Management | 20 (51.28) | 19 (48.72) |
| Assistance with Carers | 31 (79.49) | 8 (20.51) |
| Specialist Medication Management (including use of Botox and/or non-medical prescriber) | 22 (56.41) | 17 (43.59) |
| Palliative Care | 34 (87.18) | 5 (12.82) |
| Leadership and Collaboration within the MDT | 35 (89.74) | 4 (10.26) |
| Facilitation of Learning | 35 (89.74) | 4 (10.26) |
| Teaching and Assessment | 0 (0.00) | 39 (100.0) |
| Engagement with Research | 29 (74.36) | 10 (25.64) |
| Other | 5 (12.82) | 1. 87.18) |

MND, Motor Neuron Disease; NICE, National Institute for Health Care and Excellence; NHS, National Health Service; MDT, Multidisciplinary Team; n, sample size; %, percentage

**S4B Table. HCP/ Specialty Involvement in Core MDT**.

| **HCP/ Specialty** | **Yes (n (%))** | **No (n (%))** |
| --- | --- | --- |
| Neurologist | 35 (89.74) | 4 (10.26) |
| Specialist Nurse | 34 (87.18) | 5 (12.82) |
| Dietician | 31 (79.49) | 8 (20.51) |
| Physiotherapist | 29 (74.36) | 10 (25.64) |
| Occupational Therapist | 27 (69.23) | 12 (25.64) |
| Respiratory Specialist | 28 (71.79) | 11 (28.21) |
| Speech and Language | 29 (74.36) | 10 (25.64) |
| Palliative Specialist | 23 (58.97) | 16 (41.03) |
| None | 1 (2.56) | 38 (97.44) |
| Not Applicable | 0 (0.00) | 1. 100.0) |

HCP, Healthcare Professional; MDT, Multidisciplinary Team; n= sample size; %= percentage

**S4C Table. Established Relationship with NHS Services***.*

| **NHS Service** | **Yes (n (%))** | **No (n (%))** |
| --- | --- | --- |
| Clinical Psychology | 20 (51.28) | 19 (48.72) |
| Social Care | 24 (61.54) | 15 (38.46) |
| Counselling | 15 (38.46) | 24 (61.54) |
| Respiratory Ventilation Services | 36 (92.31) | 3 (7.69) |
| Gastroenterology | 32 (82.05) | 7 (17.95) |
| Orthotics | 27 (69.23) | 12 (30.77) |
| Wheelchair Services | 32 (82.05) | 7 (17.95) |
| Assistive Technology Services (e.g Augmented and alternative communication (AAC)) | 33 (84.62) | 6 (15.38) |

NHS, National Health Service; AAC, Augmented and Alternative Communication; n, sample size; %, percentage
