# Supplementary material for: An Evaluation of the United Kingdom Motor Neuron Disease Nurses and Allied Health Professionals (UK MND NAHP) Workforce: A Census
Source: PLoS One. 2025 Jul 11;20(7):e0319628. doi: 10.1371/journal.pone.0319628 (PMC12250277; doi:10.1371/journal.pone.0319628)
Supplement: S5 Table — Clinical Satisfaction Scores. MDT, Multidisciplinary Team; n, sample size; %, percentage. (DOCX) [file pone.0319628.s005.docx]

**S5 Table***.* **Clinical Satisfaction Scores**.

| **Areas of Satisfaction** | **Always (n (%))** | **Often (n (%))** | **Sometimes (n (%))** | **Never n (%))** |
| --- | --- | --- | --- | --- |
| Provision of Information and Expertise | 21 (53.85) | 18 (46.15) | 0 (0.00) | 0 (0.00) |
| Clinical Specialities | 17 (43.59) | 20 (51.28) | 1 (2.56) | 0 (0.00) |
| Collaboration and Leadership within the MDT | 20 (51.28) | 16 (41.03) | 3 (7.69) | 0 (0.00) |
| Facilitating of Learning and Education | 13 (33.33) | 21 (53.85) | 5 (12.82) | 0 (0.00) |
| Engagement with Research | 10 (25.64) | 15 (38.46) | 9 (23.08) | 5 (12.82) |
| Feelings of being valued by Healthcare Professionals within the core MDT | 27 (69.23) | 10 (25.64) | 2 (5.13) | 0 (0.00) |
| Feelings of being valued by Healthcare Professionals outside the core MDT | 15 (38.46) | 19 (48.72) | 5 (12.82) | 0 (0.00) |
| Feelings of being valued by Patients and Families and Carers | 25 (64.10) | 14 (35.90) | 0 (0.00) | 0 (0.00) |

MDT, Multidisciplinary Team; n, sample size; %, percentage
