# Supplementary material for: An Evaluation of the United Kingdom Motor Neuron Disease Nurses and Allied Health Professionals (UK MND NAHP) Workforce: A Census
Source: PLoS One. 2025 Jul 11;20(7):e0319628. doi: 10.1371/journal.pone.0319628 (PMC12250277; doi:10.1371/journal.pone.0319628)
Supplement: S6 Table — The clinical cut-off scores are grouped into three categories: 1) “Without Burnout”-Green, is between 1.00 to 2.58, 2) “At-Risk for Burnout”-Amber, the score is between 2.59 to 3.01, 3) “Likely to be Burned-Out”- Red, the score is between 3.02 to 5.00. A red score indicates that the specificity is greater than or equal to 0.90 that corresponds to a maximum misclassification of 10%. n = sample size; % = percentage. A. Burnout Assessment Tool-Core (BAT-C) Total Scores. BAT-C, Burnout Assessment Tool-Core; n, sample size; %, percentage; N/A, Not Available. B. Burnout Assessment Tool-Core (BAT-C) by Clinical HCP. BAT-C, Burnout Assessment Tool-Core; HCP, Healthcare Professional; n, sample size; %, percentage; N/A, Not Available. C. Burnout Assessment Tool-Core (BAT-C) by Non-clinical HCP. BAT-C, Burnout Assessment Tool-Core; HCP, Healthcare Professional; n, sample size; %, percentage; N/A, Not Available. D. Burnout Assessment Test-Secondary (BAT-S) Total Scores. BAT-C, Burnout Assessment Tool-Core; n, sample size; %, percentage; N/A, Not Available. E. Burnout Assessment Tool-Secondary (BAT-S) by Clinical HCP. BAT-C, Burnout Assessment Tool-Core; HCP, Healthcare Professional; n, sample size; %, percentage; N/A, Not Available. F. Burnout Assessment Tool-Secondary (BAT-S) by Non-Clinical HCP. BAT-C, Burnout Assessment Tool-Core; HCP, Healthcare Professional; n, sample size; %, percentage; N/A, Not Available (DOCX) [file pone.0319628.s006.docx]

**S6 Table. Burnout Assessment Tool.** The clinical cut-off scores are grouped into three categories: 1) “Without Burnout”-Green, is between 1.00 to 2.58, 2) “At-Risk for Burnout”-Amber, the score is between 2.59 to 3.01, 3) “Likely to be Burned-Out”- Red, the score is between 3.02 to 5.00. A red score indicates that the specificity is greater than or equal to 0.90 that corresponds to a maximum misclassification of 10%. n= sample size; %= percentage.

**S6A Table. Burnout Assessment Tool-Core (BAT-C) Total Scores.**

| **Core Burnout Categories** | **Green (n (%))** | **Amber (n (%))** | **Red (n (%))** | **N/A (n (%))** |
| --- | --- | --- | --- | --- |
| Total Score | 53 (82.81) | 6 (9.38) | 2 (3.13) | 3 (4.69) |
| Exhaustion | 46 (71.88) | 9 (14.06) | 8 (12.5) | 1 (1.56) |
| Mental Distance | 60 (93.75) | 1 (1.56) | 1 (1.56) | 2 (3.13) |
| Emotional Impairment | 48 (75.00) | 14 (21.88) | 2 (3.13) | 0 (0.00) |
| Cognitive Impairment | 50 (78.13) | 10 (15.63) | 4 (6.25) | 1. (0.00) |

BAT-C, Burnout Assessment Tool-Core; n, sample size; %, percentage; N/A, Not Available

**S6B Table. Burnout Assessment Tool-Core (BAT-C) by Clinical HCP**.

| **Core Burnout Categories** | **Green (n (%))** | **Amber (n (%))** | **Red (n (%))** | **N/A (n (%))** | **Total** |
| --- | --- | --- | --- | --- | --- |
| Total Score | 36 (92.31) | 1 (2.56) | 2 (5.13) | 0 (0.00) | **39** |
| Exhaustion | 29 (74.36) | 3 (7.69) | 7 (17.95) | 0 (0.00) | **39** |
| Mental Distance | 37 (94.87) | 1 (2.56) | 1 (2.56) | 0 (0.00) | **39** |
| Emotional Impairment | 30 (76.92) | 9 (23.08) | 0 (0.00) | 0 (0.00) | **39** |
| Cognitive Impairment | 35 (89.74) | 3 (7.69) | 1 (2.56) | 0 (0.00) | **39** |

BAT-C, Burnout Assessment Tool-Core; HCP, Healthcare Professional; n, sample size; %, percentage; N/A, Not Available

| **Core Burnout Categories** | **Green (n (%))** | **Amber (n (%))** | **Red (n (%))** | **N/A (n (%))** | **Total** |
| --- | --- | --- | --- | --- | --- |
| Total Score | 17 (68.00) | 5 (20.00) | 0 (0.00) | 3 (12.00) | **25** |
| Exhaustion | 17 (68.00) | 6 (24.00) | 1 (4.00) | 1 (4.00) | **25** |
| Mental Distance | 23 (92.00) | 0 (0.00) | 0 (0.00) | 2 (8.00) | **25** |
| Emotional Impairment | 18 (72.00) | 5 (20.00) | 2 (8.00) | 0 (0.00) | **25** |
| Cognitive Impairment | 15 (60.00) | 7 (28.00) | 3 (12.00) | 1. (0.00) | **25** |

**S6C Table. Burnout Assessment Tool-Core (BAT-C) by Non-clinical HCP***.*

BAT-C, Burnout Assessment Tool-Core; HCP, Healthcare Professional; n, sample size; %, percentage; N/A, Not Available

**S6D Table. Burnout Assessment Test-Secondary (BAT-S) Total Scores***.*

| **Green (n (%))** | **Amber (n (%))** | **Red (n (%))** | **N/A (n (%))** |
| --- | --- | --- | --- |
| 50 (78.13) | 9 (14.06) | 4 (6.25) | 1. (1.56) |

BAT-C, Burnout Assessment Tool-Core; n, sample size; %, percentage; N/A, Not Available

**S6E Table. Burnout Assessment Tool-Secondary (BAT-S) by Clinical HCP.**

| **Green (n (%))** | **Amber (n (%))** | **Red (n (%))** | **N/A (n (%))** | **Total** |
| --- | --- | --- | --- | --- |
| 30 (76.92) | 5 (12.82) | 3 (7.69) | 1 (2.56) | **39** |

BAT-C, Burnout Assessment Tool-Core; HCP, Healthcare Professional; n, sample size; %, percentage; N/A, Not Available

**S6F Table. Burnout Assessment Tool-Secondary (BAT-S) by Non-Clinical HCP***.*

| **Green (n (%))** | **Amber (n (%))** | **Red (n (%))** | **N/A (n (%))** | **Total** |
| --- | --- | --- | --- | --- |
| 20 (80.00) | 4 (16.00) | 1 (4.00) | 0 (0.00) | **25** |

BAT-C, Burnout Assessment Tool-Core; HCP, Healthcare Professional; n, sample size; %, percentage; N/A, Not Available
