# Supplementary material for: An Evaluation of the United Kingdom Motor Neuron Disease Nurses and Allied Health Professionals (UK MND NAHP) Workforce: A Census
Source: PLoS One. 2025 Jul 11;20(7):e0319628. doi: 10.1371/journal.pone.0319628 (PMC12250277; doi:10.1371/journal.pone.0319628)
Supplement: S7 Table — Burnout Assessment Tool questionnaire. A. Burnout Assessment Tool-Core (BAT-C) symptoms. B. Burnout Assessment Tool-Secondary (BAT-S) symptoms. (DOCX) [file pone.0319628.s007.docx]

**S7 Table**: **Burnout Assessment Tool questionnaire**

**S7A Table. Burnout Assessment Tool-Core (BAT-C) symptoms.**

| *Exhaustion*   1. At work, I feel mentally exhausted 2. Everything I do at work requires a great deal of effort 3. After a day at work, I find it hard to recover my energy 4. At work, I feel physically exhausted 5. When I get up in the morning, I lack the energy to start a new day at work 6. I want to be active at work, but somehow I am unable to manage 7. When I exert myself at work, I quickly get tired 8. At the end of my working day, I feel mentally exhausted and drained |
| --- |
| *Mental Distance*   1. I struggle to find any enthusiasm for my work 2. At work, I do not think much about what I am doing and I function on autopilot 3. I feel a strong aversion towards my job 4. I feel indifferent about my job 5. I’m cynical about what my work means to others |
| *Emotional Impairment*   1. At work, I feel unable to control my emotions 2. I do not recognize myself in the way I react emotionally at work 3. During my work I become irritable when things don’t go my way 4. I get upset or sad at work without knowing why 5. At work I may overreact unintentionally |
| *Cognitive Impairment*   1. At work, I have trouble staying focused 2. At work I struggle to think clearly 3. I’m forgetful and distracted at work 4. When I’m working, I have trouble concentrating 5. I make mistakes in my work because I have my mind on other things |

| *Psychological Distress*   1. I suffer from palpitations or chest pain 2. I suffer from stomach and/or intestinal complaints 3. I suffer from headaches 4. I suffer from muscle pain, for example in the neck, shoulder or back 5. I often get sick |
| --- |
| *Psychosomatic Complaints*   1. My weight fluctuates without being on a diet 2. I have trouble falling or staying asleep 3. I tend to worry 4. I feel tense and stressed 5. I feel anxious and/or suffer from panic attacks 6. Noise and crowds disturb me |

**S7B Table. Burnout Assessment Tool-Secondary (BAT-S) symptoms.**
